# Supplementary figures and images for: Impact of variants of concern on SARS-CoV-2 viral dynamics in non-human primates
Source: PLoS Comput Biol. 2023 Aug 9;19(8):e1010721. doi: 10.1371/journal.pcbi.1010721 (PMC10441782; doi:10.1371/journal.pcbi.1010721)

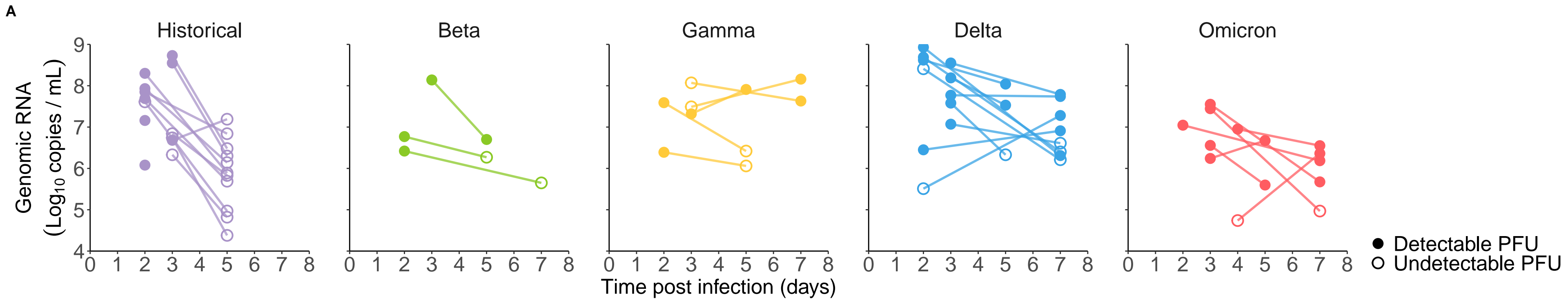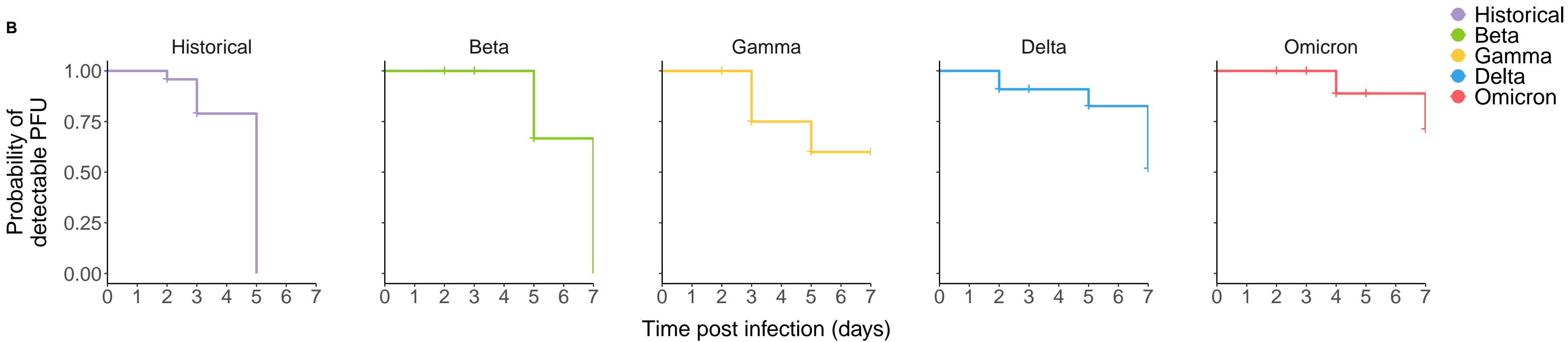

Supplement: S1 Fig — We represent the longitudinal values of genomic RNA for each individual and if the associated PFU sampe is detectable or not. (PDF) [file pcbi.1010721.s001.pdf]

Viral load (log<sub>10</sub> copies / mL)

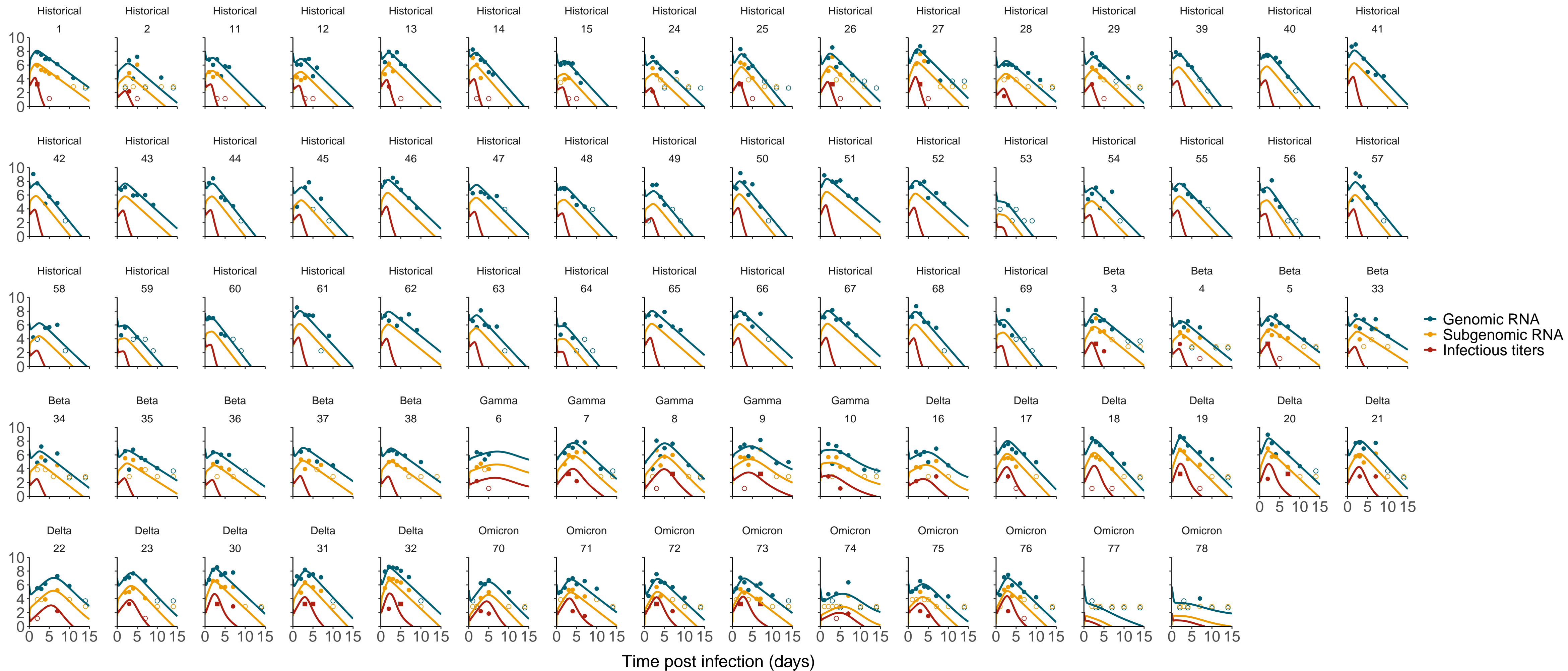

Supplement: S2 Fig — Undetectable values are represented as empty dots. Values above the upper limit of quantification are represented as squares. (PDF) [file pcbi.1010721.s002.pdf]

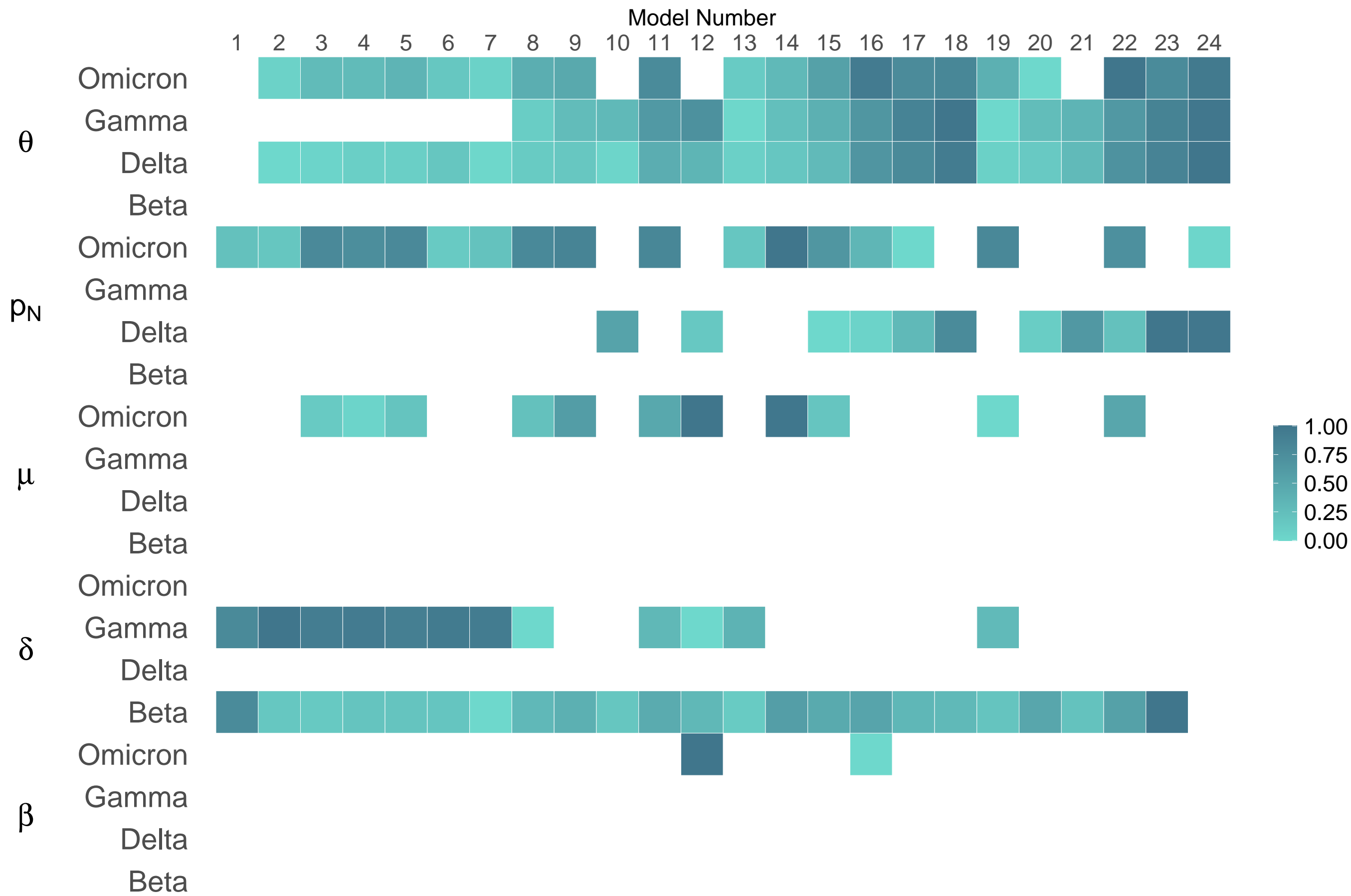

Supplement: S3 Fig — We performed a sensitivity analysis on our best model. The model IDs are represented on top, as described in S3 Table. The scale represents the magnitude of the covariate effect rescaled for each row with 0 being the minimum value and 1 the maximum. Empty tiles indicate that no covariates were selected for this variant-parameter relationship. (PDF) [file pcbi.1010721.s003.pdf]

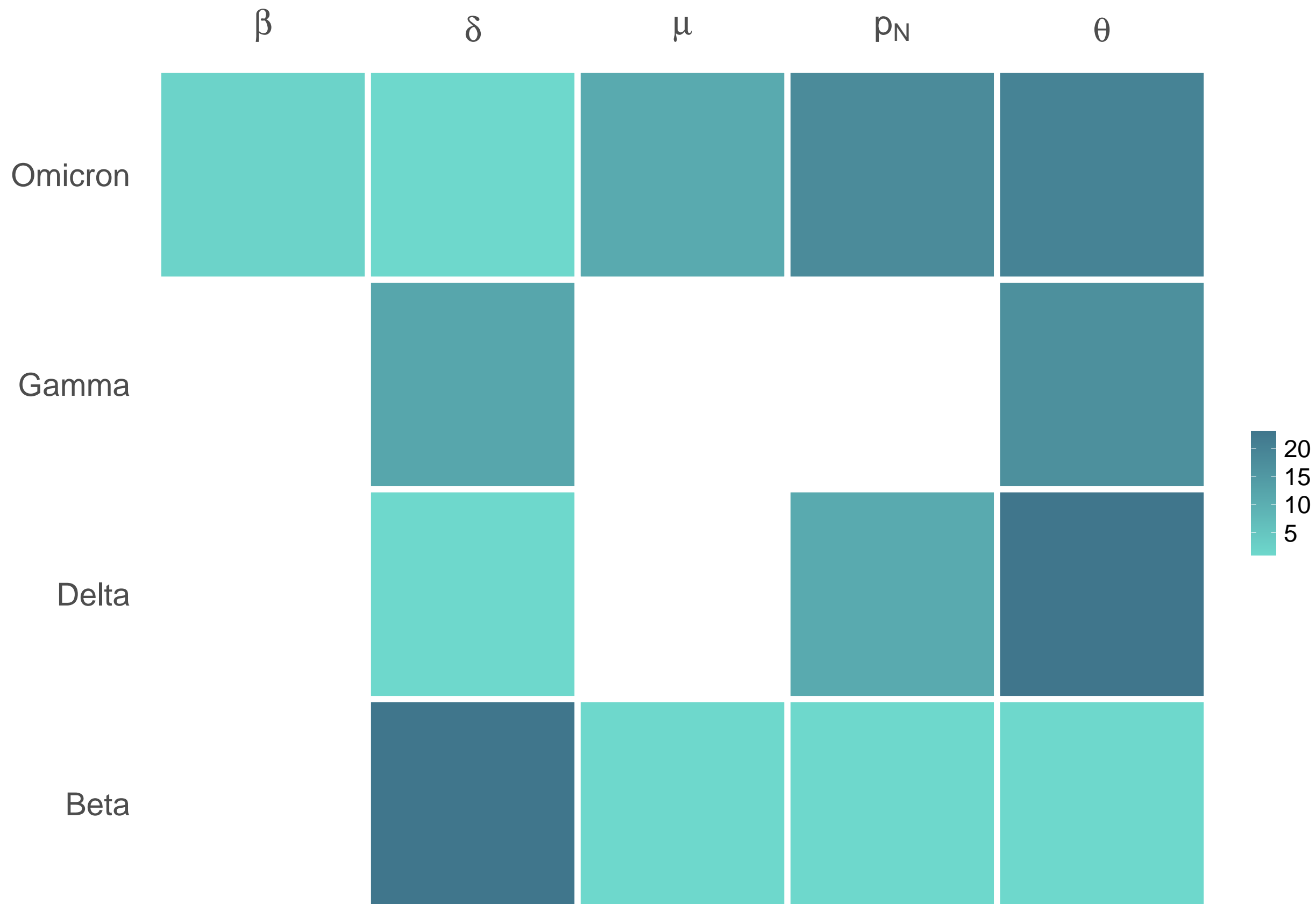

Supplement: S4 Fig — We represent the number of times a covariate was found on a variant-parameter relationship across all 24 models. Empty tiles indicate that no covariates were found for this variant-parameter relationship. (PDF) [file pcbi.1010721.s004.pdf]

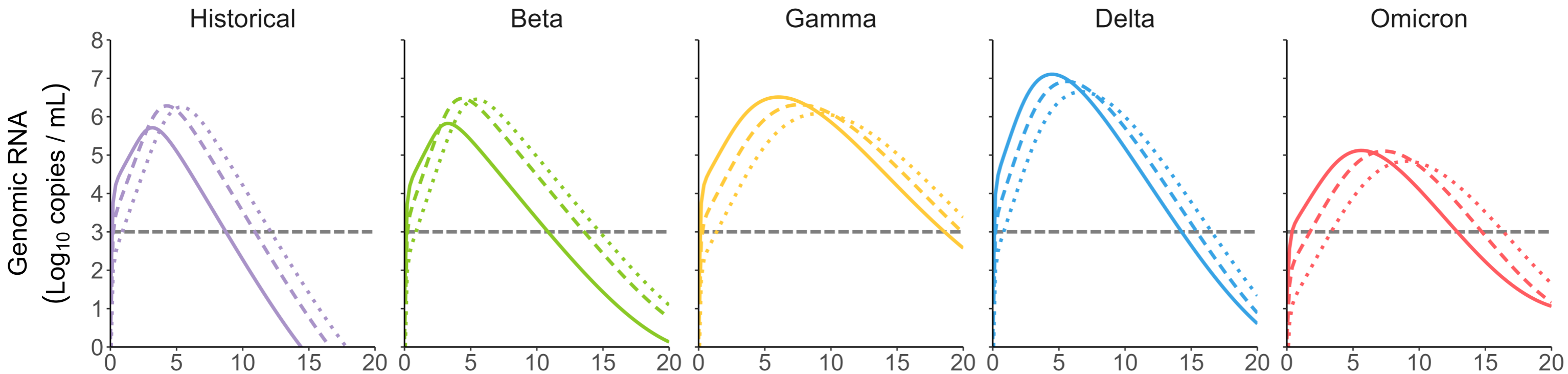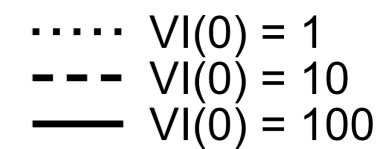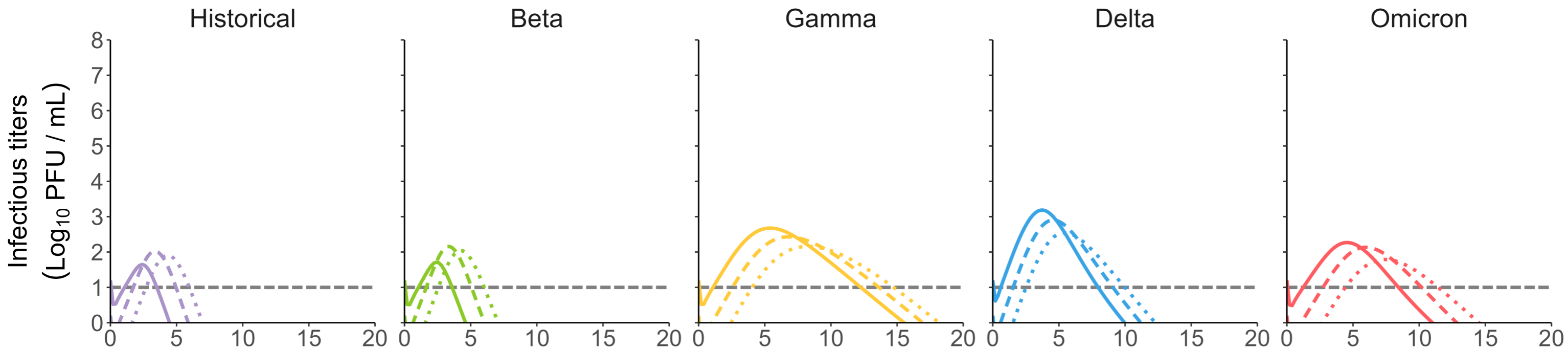

Supplement: S5 Fig — Using simulations, we sampled parameters considering both the uncertainty in the estimation and the inter-individual variability (see methods). Only the mean viral load was shown for clarity. (PDF) [file pcbi.1010721.s005.pdf]

**A**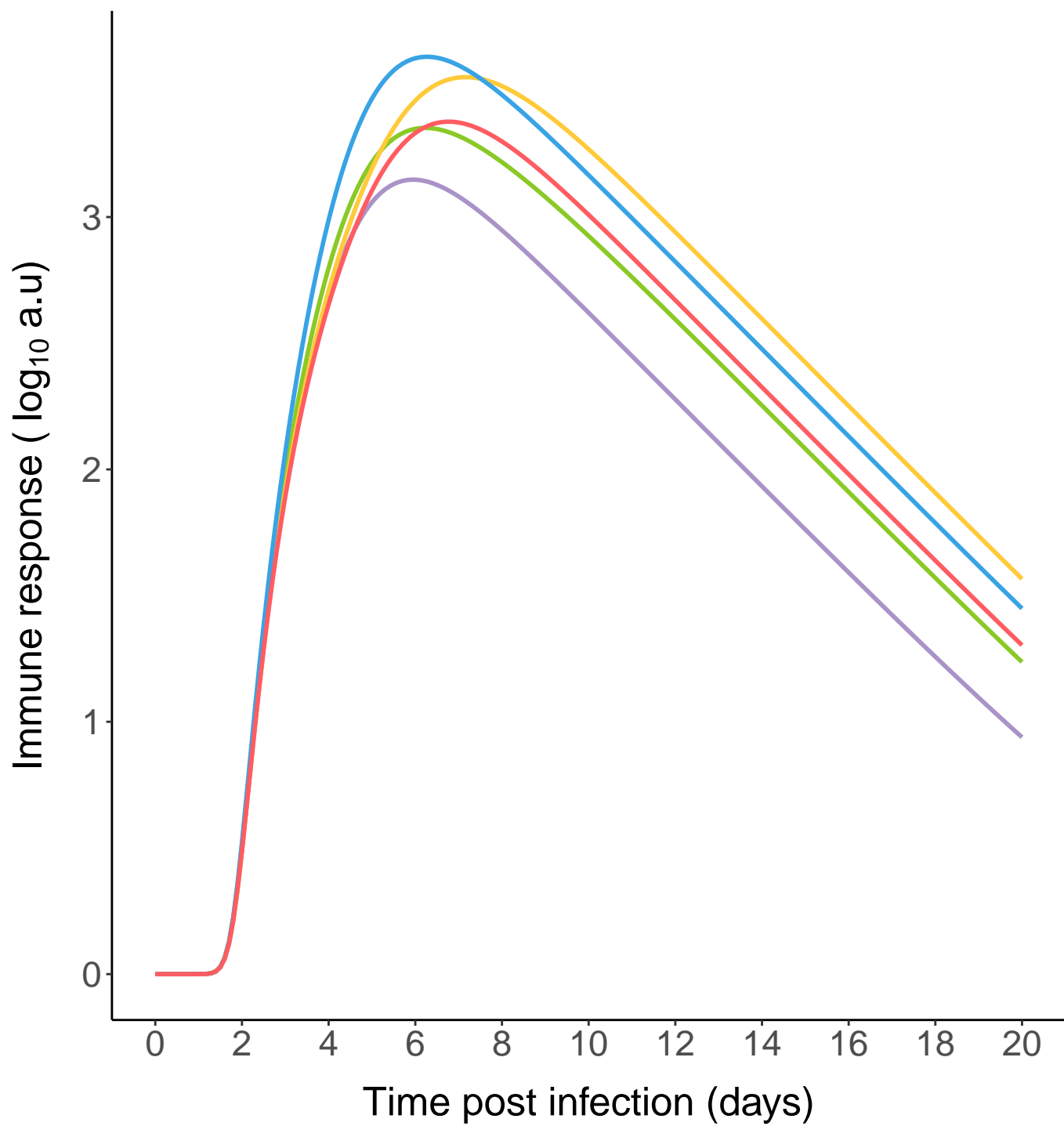**B**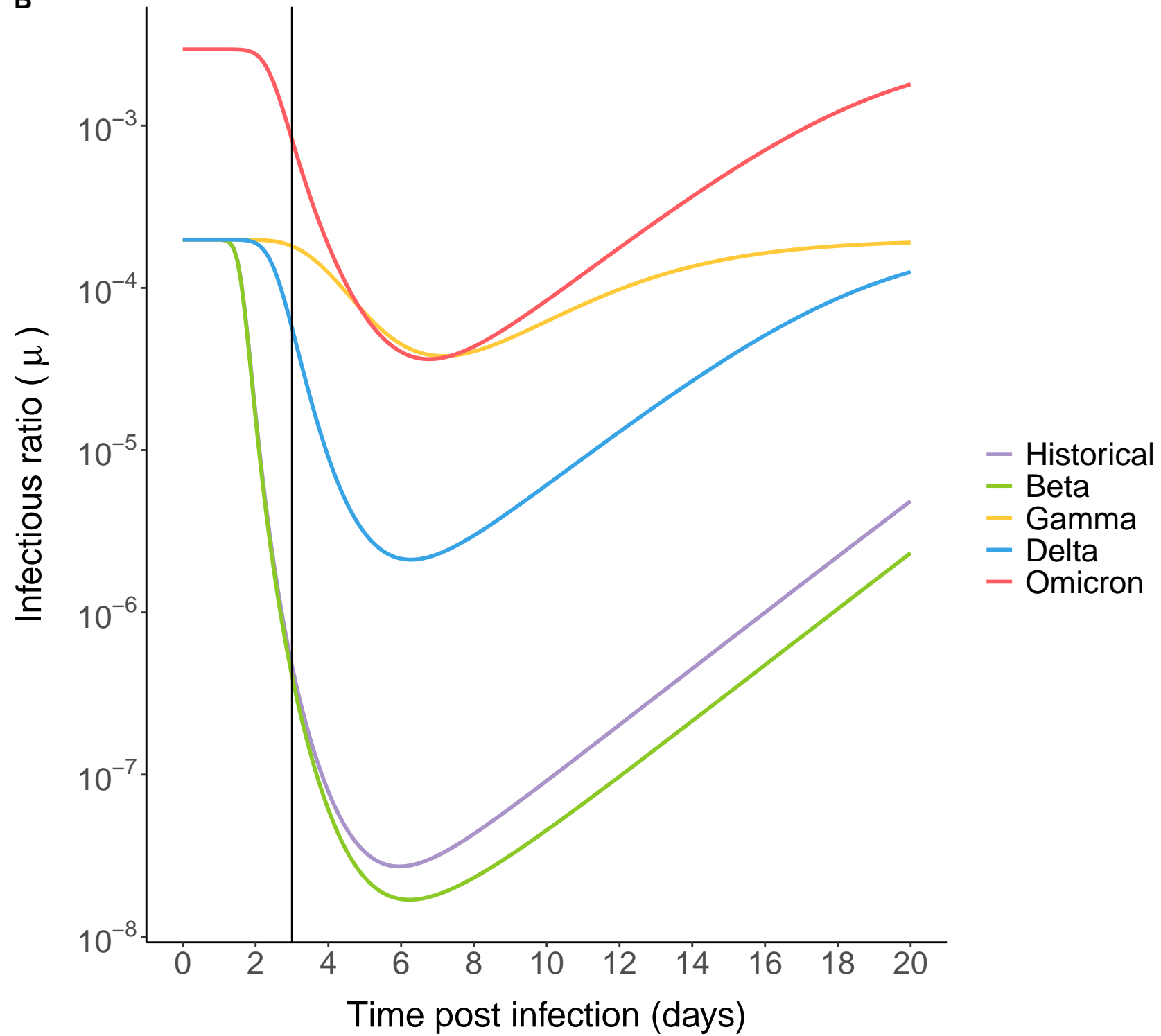

Supplement: S6 Fig — A) Median trajectory of the last compartment of our immune response B) Median trajectory of the infectious ratio parameter μ over time. We used the population parameters of our best model to simulate the median trajectory of each variant. (PDF) [file pcbi.1010721.s006.pdf]

**A**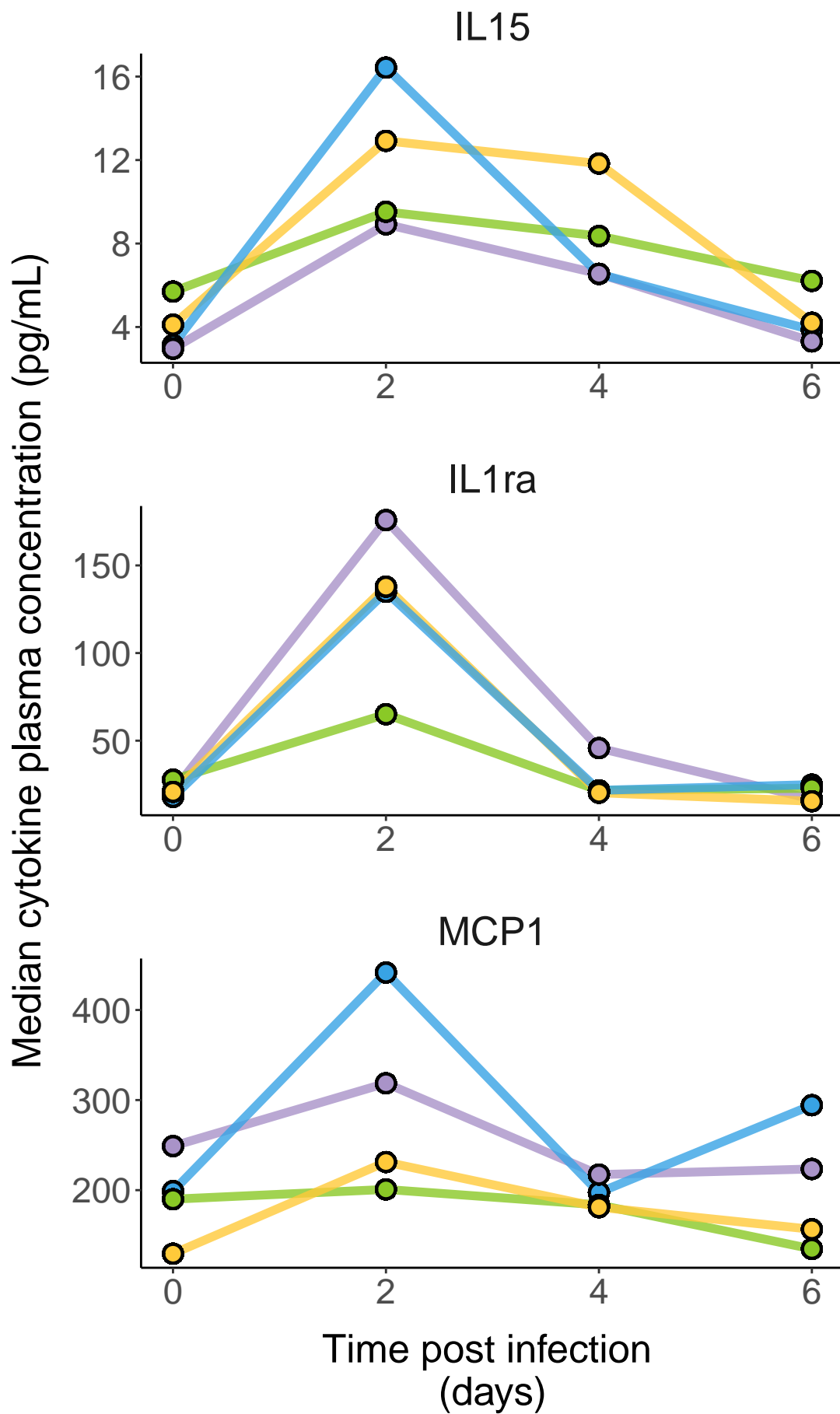**B**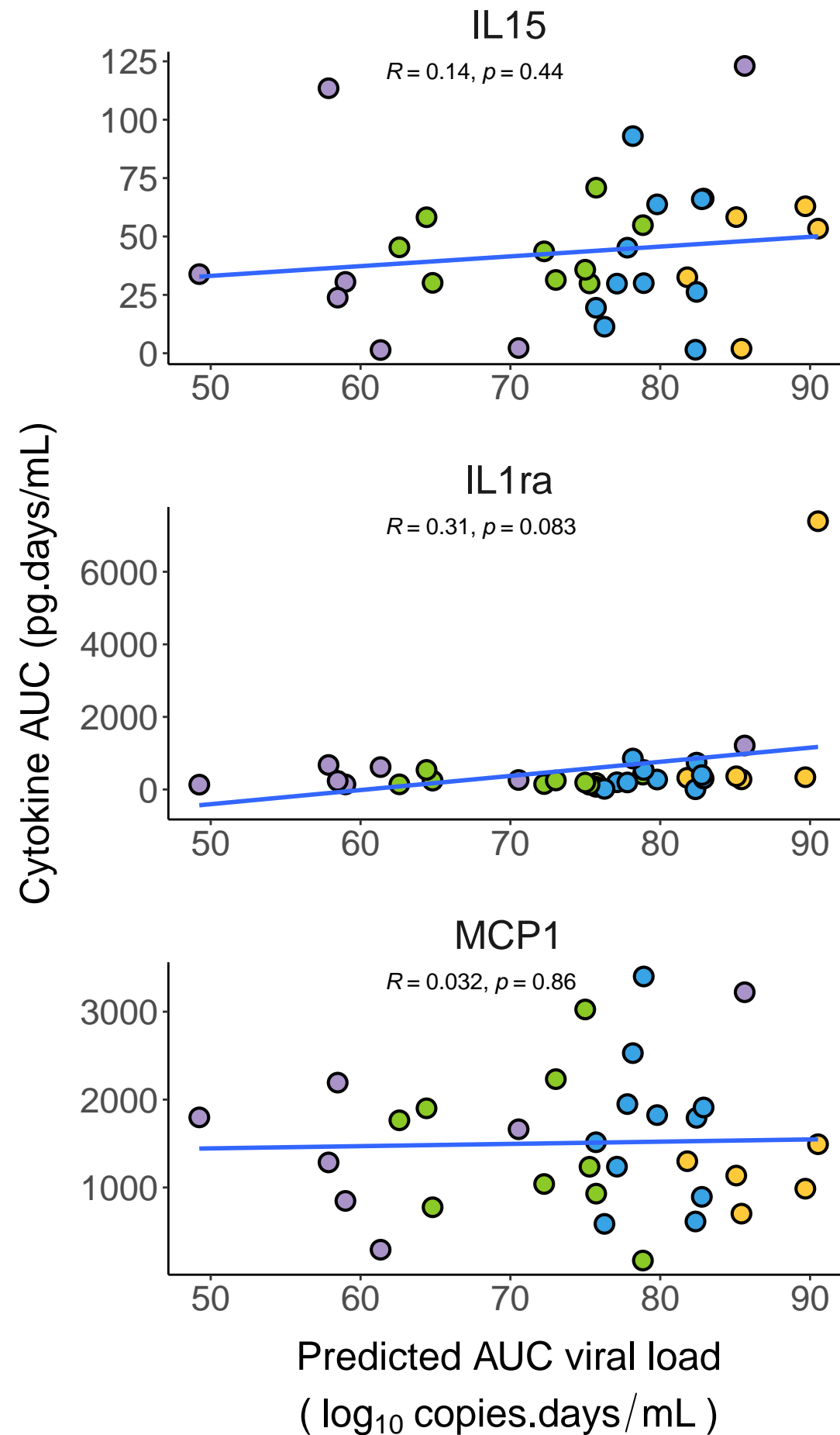**C**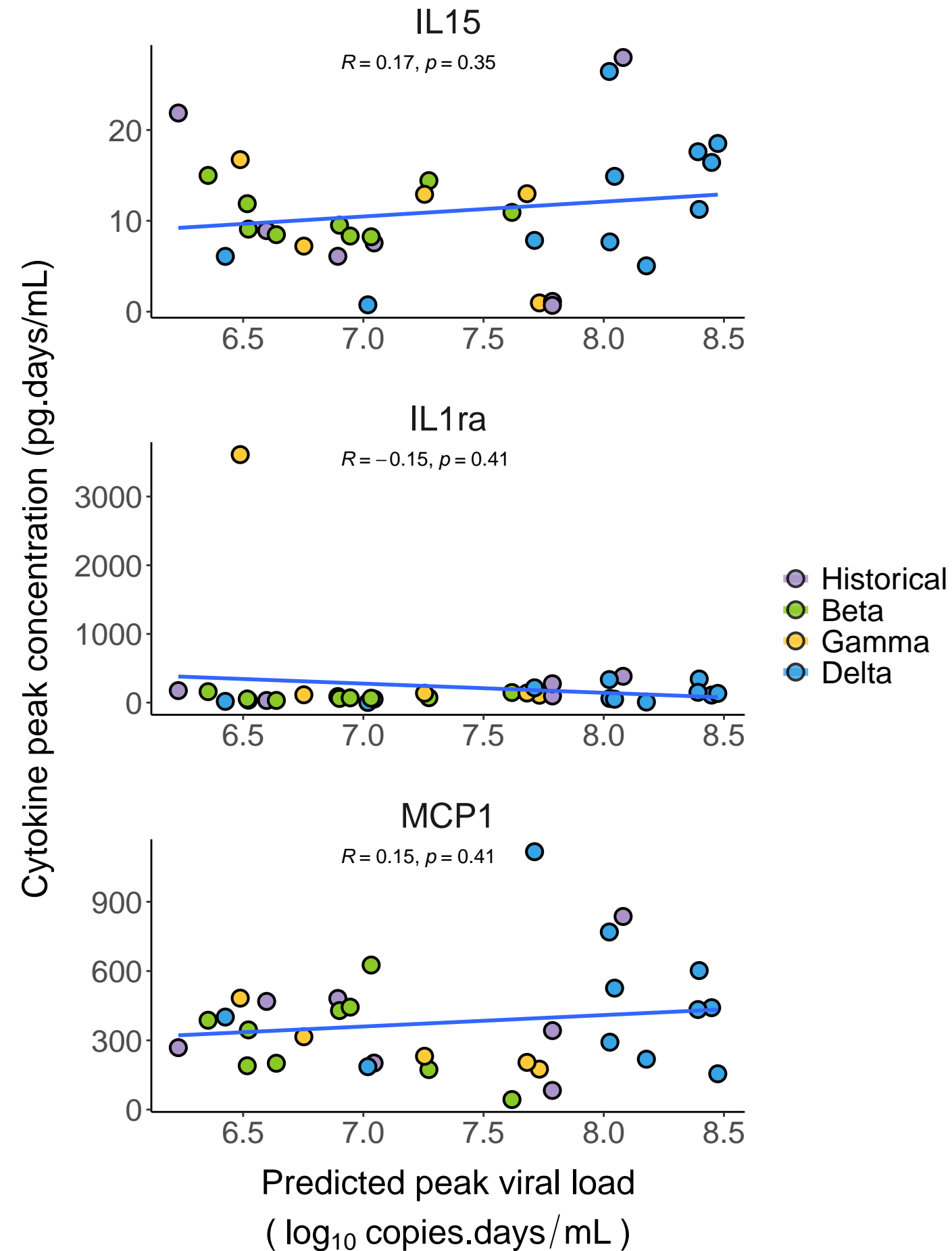

Supplement: S7 Fig — A) Median concentration of measured cytokines. B) Correlation between AUC of viral load predicted by our model and the cytokine AUC. C) Correlation between peak viral load predicted by our model and peak cytokine concentration. (PDF) [file pcbi.1010721.s007.pdf]
