# Supplementary material for: Impact of variants of concern on SARS-CoV-2 viral dynamics in non-human primates
Source: PLoS Comput Biol. 2023 Aug 9;19(8):e1010721. doi: 10.1371/journal.pcbi.1010721 (PMC10441782; doi:10.1371/journal.pcbi.1010721)
Supplement: S1 Table — Descriptive statistics of the animals calculated on the raw data. (DOCX) [file pcbi.1010721.s008.docx]

| Strains | Number of animals | Mean weight  (kg) | Mean peak viral load  (log_10_ copies/mL) | Mean peak PFU  (log_10_ PFU/mL) | Mean time to first undetectable viral load | Mean time to first undetectable PFU |
| --- | --- | --- | --- | --- | --- | --- |
| Historical | 44 | 3.7 | 7.6 | 2.3 | 8 | 4 |
| Beta | 9 | 4.9 | 7.1 | 3.2 | 10 | 6 |
| Gamma | 5 | 4.2 | 7.8 | 3 | 14 | 5 |
| Delta | 11 | 3.6 | 8.1 | 2.9 | 12 | 5 |
| Omicron | 9 | 4.6 | 6.4 | 2.4 | 12 | 7 |
