# Supplementary material for: Impact of variants of concern on SARS-CoV-2 viral dynamics in non-human primates
Source: PLoS Comput Biol. 2023 Aug 9;19(8):e1010721. doi: 10.1371/journal.pcbi.1010721 (PMC10441782; doi:10.1371/journal.pcbi.1010721)
Supplement: S2 Table — **The standard error for the R0 parameters were calculated using the delta method. (DOCX) [file pcbi.1010721.s009.docx]

| *Population parameters (unit)* | | Fixed effect (RSE%) | SD of random effect (RSE%) |
| --- | --- | --- | --- |
|  | $\beta$ (copie.d^-1^) | $1.85\times{10}^{-5} (33)$ | - |
|  | $p$ (copies.cell^-1^.d^-1^) | $9.44\times{10}^{5} (40)$ | $0.61 (17)$ |
|  | $\delta$ (d^-1^) | $1.38 (6)$ | $0.2 (20)$ |
|  | $f$ (unitless) | $1.36\times{10}^{-3} (19)$ | - |
|  | $\mu$ (unitless) | $1.98\times{10}^{-4} (47)$ | - |
|  | $\theta$ (unitless) | $0.19 (45)$ | $0.32 (103)$ |
| *Covariate model* | | Covariate effect (RSE%) | p-value |
|  | Beta on $\delta$ | -0.357 (32) | 0.00201 |
|  | Gamma on $\theta$ | 8.39 (15) | $<{10}^{-6}$ |
|  | Delta on $p$ | 0.554 (50) | 0.047 |
|  | Delta on $\theta$ | 5.49 (15) | $<{10}^{-6}$ |
|  | Omicron on $p$ | -2.82 (19) | $<{10}^{-6}$ |
|  | Omicron on $\mu$ | 2.7 (23) | $1.98\times{10}^{-5}$ |
|  | Omicron on $\theta$ | 5.04 (18) | $<{10}^{-6}$ |
| *Basic reproductive number* | | Value (RSE%)** |  |
|  | $R_{0}$ | 3.1 (19) |  |
|  | ${R_{0}}_{beta}$ | 4.5 (20) |  |
|  | ${R_{0}}_{gamma}$ | 3.1 (19) |  |
|  | ${R_{0}}_{delta}$ | 5.4 (34) |  |
|  | ${R_{0}}_{omicron}$ | 2.8 (28) |  |
| *Residual errors* | | Value (RSE%) |  |
|  | $\sigma_{Genomic RNA}$ (log_10_ copies/mL) | 0.98 (4) |  |
|  | $\sigma_{Subgenomic RNA}$(log_10_ copies/mL) | 0.89 (6) |  |
|  | $\sigma_{Infectious titers}$ (log_10_ PFU/mL) | 1.79 (14) |  |
