# Supplementary material for: Impact of variants of concern on SARS-CoV-2 viral dynamics in non-human primates
Source: PLoS Comput Biol. 2023 Aug 9;19(8):e1010721. doi: 10.1371/journal.pcbi.1010721 (PMC10441782; doi:10.1371/journal.pcbi.1010721)
Supplement: S3 Table — Using the best structural model (i.e. Model 1 including an effect on the infectious ratio) we tested several delays for the immune response to take place and performed the covariate search algorithm on all models. (DOCX) [file pcbi.1010721.s010.docx]

| Model ID | $j$  Number of compartments | $\tau$  Mean time spent | $g=\frac{j}{\tau}$  Transfer rate | Time to peak of immune compartment  (days) | BIC before COSSAC | BIC after COSSAC (ΔBIC ) |
| --- | --- | --- | --- | --- | --- | --- |
| 1 | 5 | 1 | 5 | 4 | 2451 | 2429 (-22) |
| 2 | 5 | 2 | 2.5 | 5 | 2409 | 2384 (-25) |
| 3 | 5 | 3 | 1.67 | 6 | 2405 | 2374 (-31) |
| 4 | 5 | 4 | 1.25 | 6 | 2408 | 2373 (-35) |
| 5 | 5 | 5 | 1 | 7 | 2408 | 2375 (-33) |
| 6 | 5 | 6 | 0.83 | 8 | 2409 | 2379 (-30) |
| 7 | 10 | 1 | 10 | 4 | 2432 | 2409 (-23) |
| 8 | 10 | 2 | 5 | 5 | 2409 | 2373 (-36) |
| 9 | 10 | 3 | 3.33 | 6 | 2410 | 2361 (-49) |
| 10 | 10 | 4 | 2.5 | 7 | 2411 | 2381 (-30) |
| 11 | 10 | 5 | 2 | 8 | 2413 | 2366 (-47) |
| 12 | 10 | 6 | 1.67 | 9 | 2414 | 2367 (-47) |
| 13 | 20 | 1 | 20 | 4 | 2426 | 2402 (-24) |
| 14 | 20 | 2 | 10 | 5 | 2409 | 2363 (-46) |
| 15 | 20 | 3 | 6.67 | 6 | 2411 | 2360 (-51) |
| 16 | 20 | 4 | 5 | 7 | 2414 | 2377 (-37) |
| 17 | 20 | 5 | 4 | 8 | 2416 | 2379 (-37) |
| 18 | 20 | 6 | 3.33 | 9 | 2417 | 2381 (-36) |
| 19 | 30 | 1 | 30 | 4 | 2424 | 2397 (-27) |
| 20 | 30 | 2 | 15 | 5 | 2408 | 2377 (-31) |
| 21 | 30 | 3 | 10 | 6 | 2413 | 2385 (-28) |
| 22 | 30 | 4 | 7.5 | 7 | 2417 | 2363 (-54) |
| 23 | 30 | 5 | 6 | 8 | 2419 | 2380 (-39) |
| 24 | 30 | 6 | 5 | 9 | 2420 | 2393 (-27) |
