# Supplementary material for: Impact of variants of concern on SARS-CoV-2 viral dynamics in non-human primates
Source: PLoS Comput Biol. 2023 Aug 9;19(8):e1010721. doi: 10.1371/journal.pcbi.1010721 (PMC10441782; doi:10.1371/journal.pcbi.1010721)
Supplement: Attachment — Submitted filename: AnswerToReviewers - Plos.docx [file pcbi.1010721.s011.docx]

# Impact of variants of concern on SARS-CoV-2 viral dynamics in non-human primates

Aurélien Marc^1^, Romain Marlin^2^, Flora Donati^3,4^, Mélanie Prague^5,6^, Marion Kerioui^1^, Cécile Hérate^2^, Marie Alexandre^5,6^ , Nathalie Dereuddre-bosquet^2^, Julie Bertrand^1^, Vanessa Contreras^2^, Sylvie Behillil^3,4^, Pauline Maisonnasse^2^, Sylvie Van Der Werf ^3,4^, Roger Le Grand^2^, Jérémie Guedj^1^

1 Université Paris Cité, INSERM, IAME, UMR 1137, 75018, Paris, France

2 Université Paris-Saclay, Inserm, CEA, Center for Immunology of Viral, Auto-immune, Hematological and Bacterial diseases (IMVA-HB/IDMIT), Fontenay-aux-Roses and Le Kremlin-Bicêtre, Paris, France

3 National Reference Center for Respiratory Viruses, Institut Pasteur, 28 rue du Docteur Roux, 75015 Paris, France.

4 Molecular Genetics of RNA Viruses Unit, Institut Pasteur, UMR3569, CNRS, Université de Paris, 28 rue du Docteur Roux, 75015 Paris, France.

5 Inria Bordeaux Sud-Ouest, Inserm, Bordeaux Population Health Research Center, SISTM Team, UMR 1219, University of Bordeaux, Bordeaux, France

6 Vaccine Research Institute, Créteil, France

**Responses to referees and editor**

We thank the reviewers and the reviewing editor for their insightful comments.

The main changes are as follows:

- We have added one candidate model of immune response from Guedj et al. (PMID: 33536313), the refractory model.
- As per reviewer 1 comments, we added a discussion of immunity and how our results relate to current knowledge on variant of concern (VoC) immune escape and mutations.
- We performed additional simulations including lower dose inoculum to assess its impact on our results.

Please find our answers below each comment.

Reviewer #1:

The authors used longitudinal viral load data from macaques infected with SARS-CoV-2 to study the viral dynamics of some SARS-CoV-2 variants. They analyzed the data using several hypothesized mathematical models to understand how the virus infection dynamics differ among the major variants of concern (VOCs). They found that Omicron variants had a high infectious virus production rate but a low peak viral load, and they discussed the relationship between these features and the high infectivity and low pathogenicity of Omicron variants. However, the current version of the manuscript may lack a discussion of immunity, despite the fact that the conclusion of the research is closely related to the host immune response. Also, the mathematical modeling and data analysis are need to be update. Therefore, I recommend resubmitting the revision with the following concerns addressed:

1. First of all, the data for TCID50 is extremely limited, with only two time points at maximum and many undetectable values, particularly in the late phase. Despite using these limited datasets, the authors concluded that the best model among four hypothetical mechanisms of action by immune cells is a model targeting the infectious ratio through immune response (Model 1). Apart from BIC, they need to explain how this model is validated from immunological point of view rather than statistical point of view.
We agree with the reviewer that infectious titers data are limited, which is due to the fact that measuring infectious titers is a tedious and costly procedure, feasible only in a secure BSL-3 lab. This is why the majority of data published until now focus only on genomic viral load without measuring the infectious titers. Albeit limited, the strength of our study is to measure infectious titers longitudinally at two distinct time points in nearly 40 animals. Using these data, one can clearly observe that all infectious titers samples become undetectable at day 5 post infection for the historical variant despite having relatively high viral load (S1 Fig. A). In contrast, the kinetics of infectious virus is different with the variants, that remain infectious for longer period of time, often beyond day 7. This is now clearly exemplified in the new S1 Fig. B.

Also, we agree that a more biological discussion on immune response was lacking. Per your comment, we have added the following paragraph in the discussion:

*“**We developed a model that incorporates the effect of an antigen-mediated immune response that reduces the ratio of infectivity, noted mu, via a non-linear sigmoid-type function. The model predicts that the immune response reaches a its peak value at day 5 (S6 Fig). How can we relate this compartment to biological markers? The predicted kinetics is too precocious to be related to the adaptative response, since no antibody or T-cells response is measured until day 10 post infection in this experimental model* [1–4]*. This would rather favor an effect of the innate immune response, which is the first line of defense of the host during an infection. For instance the interferon response elicit strong protection against viruses infections* [5] *by activating hundreds of interferon stimulated genes (ISGs) which have antiviral, antiproliferative and immunomodulatory functions* [6]*. Further SARS-CoV-2 variants have been shown to exhibits mutations in nucleocapsid, membrane and nonstructural proteins that antagonize IFN signaling in cells* [7,8] *and reduce its antiviral effect* [4,9]*. This is captured in our model through the parameter*$\theta$*, which is higher in SARS-CoV-2 variants and therefore limits the effect of IFN in reducing the ratio of infectious particles. Nonetheless it should be recognized that this hypothesis remains to be validated by experimental data, as IFN was not measured in this study. Other cytokines that were available (eg., MCP1, IL15 and IL1RA) showed no correlation with viral kinetics (S7 Fig), and peaked earlier than predicted by our model (typically at day 2 post infection).*

2. In Guedj et al. (PMID: 33536313), they proposed “refractory model” and use it for analyzing the longitudinal VL data. What is the reason that they do not propose the refractory model here for the candidate model. In fact, there are a hundred of different mathematical model considering different types of immune responses?

We agree with the reviewer and have now added the refractory model to the candidate models. The decrease in target cell becoming refractory to infection is then written as follows:

$$\frac{dT}{dt}=-\beta TV_{I}-\varphi\frac{F_{20}}{F_{20}+\theta}T$$

We have updated the model selection procedure however, our results remain unchanged (Table 1).

3. They chose the Model 1 by data fitting to “historical variant” and then a covariate search algorithm was used to find the most likely VOC associated effects. In general, because SARS-CoV-2 evolves to escape host immune response including vaccine-elicited immune responses, the mechanism of action by immune system may be different. That is, even if the best model for historical variants is Model 1, best models for other VOCs might be different model (e.g., Model 2, Model 3, Model 4). In addition, they need to explain how VOC-specific mutations in sequences is corresponding to an ability to escape from immune responses (i.e., the mutations on VOCs affect the function of the infectious virus production).

We chose to use all data available, regardless of variants, to perform our model selection and covariate search algorithm, and hence maximize the statistical power to distinguish different models. However, we agree with the reviewer that this assumes that the same model applies to all variants. We have better described our procedure in the methods: *“[…] Models were estimated using all data (historical and variants) and compared based on BIC, precision of the estimation and goodness of fit. A covariate search algorithm was then used on the selected model (see below) to identify VoC-specific effects.”*

We also acknowledge the limitation of this strategy in the discussion: *“[…]* *As our model selection process was performed using all data available, this assumes that the same model applies to all variants, which may not be true. Nonetheless, data limitation did not allow to perform model selection on each variant separately.”*

Regarding your comment on the biological ground of this model, we have added a full paragraph related to this question (see point 1 above).

4. While estimated parameter on mu (i.e., the ratio of infectious virus), there is no discussion on theta (i.e., the amount of immune effector needed to reduce by 50% infectious ratio). In terms of data fitting, independently estimation mu and theta are difficult because these two parameters have a complementary role for virus infection dynamics (i.e., distinguish [small mu and large theta] and [large mu and small theta] is difficult).

The reviewer is correct both parameter mu and theta are correlated. Here, the estimation of both parameters is made possible by the fact that both infectious and total virus are measured, in the inoculum and during the follow up. It is this information that makes it possible to estimate mu and theta parameters with good precision, as reflected by the relative standard errors of estimated parameters (RSE of both parameters < 50%).

In addition, and to reinforce the plausibility of our results, similar order of magnitude for mu was previously found in Gonçalves et al. [10] and several other studies have estimated that there was about 1 infectious particle for 1000 to 10 000 copies of viral RNA [11].

The reviewer is also correct that the theta parameter has no biological ground and is purely phenomenological which limits its biological interpretation.

We added this in the discussion: *“[…]one should acknowledge that the amount of infectious virus remains limited, both in terms of number of points and in the capability to measure it precisely. Although the data available here allowed us to estimate all parameters with good precision, including the changes in infectivity over time, some aspects of the model (such as the effect of the immune system on infectivity, noted*$\theta$*) remain phenomenological.”*

5. They found “All variants except beta have shown an effect on the antigen-mediated response, greatly reducing its impact on viral kinetics. As the effect of the antigen-mediated response was impaired, the infectious ratio remains higher than what was observed for the historical strain leading to more infectious particles produced over longer periods of time.” and explained “This is in line with numbers of studies showing the immune escape capabilities of those variants”. The authors have to discuss how their conclusion based on estimated parameters for immune escape (i.e., changes on mu and theta) are consistent with the current knowledge on SARS-CoV-2 immune escape because, to date, there are numerous advances on understanding immune response to different VOCs.

We addressed the reviewer comment by adding a discussion of immunity in the paper (see point #1).

6. The authors have not included a simulation of the host immune response by their mathematical model. In order to validate their mathematical model and the estimated parameters, they should present a simulation of the host immune response to VOCs and discuss how it compares to recent studies on immune responses to VOCs.

We addressed the reviewer comment by adding a discussion of immunity in the paper (see point #1).

We have added a simulations of the host immune response in the supplementary showing that it attains its peak value at day 5 post infection and that the kinetics are similar between each variant. (S6 Fig).

7. The assumptions about the initial values are unclear. What do "0.1" and "V_NI(0)_i-V_I(0)_i" represent? It is not clear if it is appropriate to perform arithmetic operations with these values, given that they have different units.

We thank reviewer for this comment as this wasn’t clear enough. We have clarified this in the methods: *“[…]* *Animals are infected using both an intranasal and intratracheal route using 90% of the initial inoculum in the trachea and 10% in the nose* [12]*. We take this into account by adding a 0.1 factor in our initial conditions.*

*[…], where* $V_{I}\left( 0 \right)_{i}$ *is the number of infectious virus of subject* $i$ *(obtained assuming that 1 PFU = 1 infectious particle),* $V_{NI}\left( 0 \right)_{i}$ *is the number of non-infectious virus (obtained by the difference between the total number of RNA copies and the number of infectious titers in the inoculum)* *and* $h_{i}$ *the proportion of the inoculum actively initiating the infection.”*

8. It is not clear what the purpose of fitting the subgenomic RNA is or if it improves the parameter estimation.

We agree with reviewer that in our context the addition of subgenomic RNA has little impact on the precision of parameter estimation. Nonetheless, adding subgenomic RNA is important for other purposes, such as evaluating the efficacy of antiviral treatment as it allows to differentiate RNA copies produced *de novo* from the administered inoculum [12,13]. We therefore believe that for the sake of consistency it is better to analyze both genomic and subgenomic RNA.

We nevertheless provide for your consideration the results obtained without sgRNA and show that they are identical.

|  | | Best model | | Best model without sgRNA | |
| --- | --- | --- | --- | --- | --- |
| *Population parameters (unit)* | | Fixed effect (RSE%) | SD of random effect (RSE%) | Fixed effect (RSE%) | SD of random effect (RSE%) |
|  | $\beta$ (copie.d^-1^) | $1.85\times{10}^{-5} (33)$ | - | $2.5\times{10}^{-5}$ (34) | - |
|  | $p$ (copies.cell^-1^.d^-1^) | $9.44\times{10}^{5} (40)$ | $0.61 (17)$ | $7.79\times{10}^{5}$ (20) | $0.6 (18)$ |
|  | $\delta$ (d^-1^) | $1.38 (6)$ | $0.2 (20)$ | $1.37 (5)$ | $0.21 (26)$ |
|  | $f$ (unitless) | $1.36\times{10}^{-3} (19)$ | - | - | - |
|  | $\mu$ (unitless) | $1.98\times{10}^{-4} (47)$ | - | $1.6\times{10}^{-4} (39)$ | - |
|  | $\theta$ (unitless) | $0.19 (45)$ | $0.32 (103)$ | $0.31 (55)$ | $0.31 (127)$ |
| *Covariate model* | | Covariate effect (RSE%) | p-value | Covariate effect (RSE%) | p-value |
|  | Beta on $\delta$ | $-0.357 (32)$ | $0.002$ | $-0.36 \left( 37 \right)$ | $0.0067$ |
|  | Gamma on $\theta$ | $8.39 (15)$ | $<{10}^{-6}$ | $8.7 (14)$ | ${<10}^{-6}$ |
|  | Delta on $p$ | $0.554 (50)$ | $0.047$ | $0.6 (52)$ | $0.053$ |
|  | Delta on $\theta$ | $5.49 (15)$ | $<{10}^{-6}$ | $6.5 (24)$ | $4.5\times{10}^{-5}$ |
|  | Omicron on $p$ | $-2.82 (19)$ | $<{10}^{-6}$ | $-2.8 \left( 18 \right)$ | $<{10}^{-6}$ |
|  | Omicron on $\mu$ | $2.7 (23)$ | $1.98\times{10}^{-5}$ | $2.5 (23)$ | $1.2\times{10}^{-5}$ |
|  | Omicron on $\theta$ | $5.04 (18)$ | $<{10}^{-6}$ | $5.7 (18)$ | $<{10}^{-6}$ |

9. As they also mentioned in Discussion section, the initial virus dose is much higher than natural infection, so the estimation of the parameters related to the growth phase of virus production may not be accurate. Especially, this is expected to have affected the time-to-peak and duration of virus shedding of estimated viral dynamics. The effect of the initial virus dose on parameter estimation should be discussed.

In non-human primate studies, animals are inoculated with large quantities of virus to ensure successful infection and repeatability of the results. However, this is indeed, a limitation, in particular to estimate the initial phase of the infection, as we acknowledged in the initial version of the manuscript. Despite this limitation we still estimate an intra-host reproduction number ($R_{0}$) consistent with previous estimation [10] and our prediction obtained assuming a lower inoculum in the model is consistent with what is observed in humans, with a peak viral load at day 5 post infection [14,15].

We have added the following in the discussion: *“**First, in this experimental model the inoculated dose is extremely high compared to a human infection, which is typically initiated with 1-10 infectious particles [19].* *This inherent limitation to NHP studies leads to a rapid saturation of target cells making it difficult to accurately estimate the early phase of infection. Here, however, our prediction obtained assuming a lower inoculum is in line with what was observed in humans, with a peak viral load around 5 days post infection* [14,15]*.”*

10. The authors assumed that the correction for the amount of measured viral load due to the structure of the animal's nasal cavity is 20% without providing any justification or references. This assumption should be supported with references.

Reviewer 1 is correct that the parameter $h$ cannot be experimentally measured, and could vary across animals due to the size of the nasal cavity. To address this limitation, we incorporated a variability on $h$ via random effects and we tested several values for $h$ in our final model. The model providing the lowest BIC was selected. In case of similar BIC, the model providing lowest residual standard error was selected.

We added additional information in our methods regarding this parameter: *“[…]* *We introduced a parameter* $h$ *representing the proportion of the inoculum that arrive on the site of infection. Because this parameter cannot be reliably estimated or experimentally measured, a profile likelihood approach was used, testing values ranging from 10% to 90%.”*

We nevertheless provide for your consideration the results obtained with different values of $h$:

|  | | Estimates (RSE%) | | | | | | | | |
| --- | --- | --- | --- | --- | --- | --- | --- | --- | --- | --- |
|  | | Value of h (%) | | | | | | | | |
| *Covariate model* | | 10 | 20 | 30 | 40 | 50 | 60 | 70 | 80 | 90 |
|  | Beta on $\delta$ | -0.36 (34) | -0.36 (32) | -0.37 (32) | -0.39 (32) | -0.4 (30) | -0.37 (31) | -0.38 (34) | -0.39 (31) | -0.38 (37) |
|  | Gamma on $\theta$ | 8.23 (12) | 8.39 (15) | 8.38 (14) | 8.47 (13) | 8.18 (16) | 7.5 (22) | 7.6 (24) | 8.21 (46) | 6.9 (43) |
|  | Delta on $p$ | 0.5 (54) | 0.55 (50) | 0.55 (47) | 0.51 (49) | 0.51 (49) | 0.57 (44) | 0.61 (46) | 0.61 (47) | 0.69 (39) |
|  | Delta on $\theta$ | 6.38 (17) | 5.49 (15) | 5.45 (15) | 5.58 (17) | 5.61 (17) | 5.13 (24) | 5.05 (26) | 5.63 (60) | 4.62 (26) |
|  | Omicron on $p$ | -3.06 (59) | -2.82 (19) | -3.04 (24) | -3.26 (12) | -3.23 (14) | -3.1 (16) | -3.02 (17) | -3.3 (26) | -3.36 (61) |
|  | Omicron on $\mu$ | 2.95 (71) | 2.7 (23) | 2.96 (31) | 3.22 (12) | 3.16 (16) | 3.08 (16) | 2.98 (18) | 3.31 (36) | 3.45 (71) |
|  | Omicron on $\theta$ | 5.79 (57) | 5.04 (18) | 5.54 (19) | 5.79 (20) | 6.03 (18) | 5.05 (37) | 4.99 (49) | 5.76 (14) | 5.12 (35) |
|  | -2LL | 2277 | 2278 | 2280 | 2283 | 2286 | 2287 | 2290 | 2295 | 2301 |
|  | BIC | 2360 | 2360 | 2363 | 2366 | 2368 | 2370 | 2373 | 2378 | 2384 |

11. In Fig S2, cases 77 and 78 had poor fitting results because there were not enough detectable points. This may significantly impact the parameter estimation for Omicron variants and the overall features of viral dynamics. Is there a specific reason why they were not excluded?

Although these animals do not present any detectable viral load, we prefer to use all information available to avoid any risk of bias.

Nonetheless, we present for your consideration the results obtained without these animals and show that they remain unchanged:

|  | | Best model | | Best model without 2 omicron infected animals | |
| --- | --- | --- | --- | --- | --- |
| *Population parameters (unit)* | | Fixed effect (RSE%) | SD of random effect (RSE%) | Fixed effect (RSE%) | SD of random effect (RSE%) |
|  | $\beta$ (copie.d^-1^) | $1.85\times{10}^{-5} (33)$ | - | $1.99\times{10}^{-5} (51)$ | - |
|  | $p$ (copies.cell^-1^.d^-1^) | $9.44\times{10}^{5} (40)$ | $0.61 (17)$ | $9.81\times{10}^{5}$ (22) | $0.5 (18)$ |
|  | $\delta$ (d^-1^) | $1.38 (6)$ | $0.2 (20)$ | $1.38 (5)$ | $0.2 (21)$ |
|  | $f$ (unitless) | $1.36\times{10}^{-3} (19)$ | - | $1.32\times{10}^{-3} (20)$ | - |
|  | $\mu$ (unitless) | $1.98\times{10}^{-4} (47)$ | - | $1.6\times{10}^{-4} (47)$ | - |
|  | $\theta$ (unitless) | $0.19 (45)$ | $0.32 (103)$ | $0.22 (69)$ | $0.61 (122)$ |
| *Covariate model* | | Covariate effect (RSE%) | p-value | Covariate effect (RSE%) | p-value |
|  | Beta on $\delta$ | $-0.357 (32)$ | $0.002$ | $-0.37 \left( 33 \right)$ | $0.002$ |
|  | Gamma on $\theta$ | $8.39 (15)$ | $<{10}^{-6}$ | $7.9(19)$ | ${<10}^{-6}$ |
|  | Delta on $p$ | $0.554 (50)$ | $0.047$ | $0.5 (43)$ | $0.01$ |
|  | Delta on $\theta$ | $5.49 (15)$ | $<{10}^{-6}$ | $5.5 (29)$ | $5.7\times{10}^{-4}$ |
|  | Omicron on $p$ | $-2.82 (19)$ | $<{10}^{-6}$ | $-2.4 \left( 22 \right)$ | $<{10}^{-6}$ |
|  | Omicron on $\mu$ | $2.7 (23)$ | $1.98\times{10}^{-5}$ | $2.8 (19)$ | $<{10}^{-6}$ |
|  | Omicron on $\theta$ | $5.04 (18)$ | $<{10}^{-6}$ | $5.2 (35)$ | $0.0043$ |

Reviewer #2:

The study investigates virus and immune dynamics in 78 macaques challenged with the original strain and four variants of concern with the aim of determining each strain viral-immune-specific characteristics. The study is clearly reported, the results are interesting, and the scope is appropriate for the journal. I have a few concerns/suggestions that I will detail below

1. Please add a parameter table and include initial condition. Some IC are listed throughout the text but I could not find initial conditions for F1, …, F20.

The parameter table can be found in the supplementary as S2 Table Estimates of the population parameter and covariate effects for the best model. We have also clarified the initial conditions for the different immune compartments in the methods: *“[…]* *All immune response compartments were set to 0 at* $t=0$*.”*

2. I am surprised about the choice of VI(0). While I understand that it was set at 10 to match human studies, it is very low compared to the inoculum RNA. Do the results change if VI(0) is varied? Given that the study investigates the differences in VoC, it is possible that the initial inoculum is a factor as well. Maybe fewer VI start infection in certain variants. Can you add VI(0) as an unknown in the data fitting?

We have added simulations with 100 and 1 infectious particles. The results obtained with 1 pfu are identical to those observed with 10 pfu, while 100 pfu predicts a more rapid kinetics (S5 Fig).

Regarding the question on the inoculum, we have clarified that both the quantity of infectious particles and the total amount of RNA were measured experimentally in all animals. In addition, a parameter $h$ is introduced to account for variations in the actual quantity of virus that reaches the nasal cavity, and an effect of VoC on this parameter was tested (see methods).

3. Can you explain the reasoning for equating I2 with the subgenomic data. Why not I1+I2?

The reviewer is correct that both models could be tested. As the half-life of $I_{1}$ is short (4h) the vast majority of infected cells are productively infected, making the contribution of $I_{1}$ to sgRNA negligible.

For your consideration, we provide below the results obtained with this model.

|  | | Best model | | Best model with different formula for subgenomic  $sgRNA=f*p*(I_{1}+I_{2})$ | |
| --- | --- | --- | --- | --- | --- |
| *Population parameters (unit)* | | Fixed effect (RSE%) | SD of random effect (RSE%) | Fixed effect (RSE%) | SD of random effect (RSE%) |
|  | $\beta$ (copie.d^-1^) | $1.85\times{10}^{-5} (33)$ | - | $1.79\times{10}^{-5} (38)$ | - |
|  | $p$ (copies.cell^-1^.d^-1^) | $9.44\times{10}^{5} (40)$ | $0.61 (17)$ | $9.94\times{10}^{5} (51)$ | $0.6 (16)$ |
|  | $\delta$ (d^-1^) | $1.38 (6)$ | $0.2 (20)$ | $1.39 (5)$ | $0.2 (28)$ |
|  | $f$ (unitless) | $1.36\times{10}^{-3} (19)$ | - | $1.1\times{10}^{-3} (35)$ | - |
|  | $\mu$ (unitless) | $1.98\times{10}^{-4} (47)$ | - | $1.88\times{10}^{-4} (35)$ | - |
|  | $\theta$ (unitless) | $0.19 (45)$ | $0.32 (103)$ | $0.22 (49)$ | $0.47 (204)$ |
| *Covariate model* | | Covariate effect (RSE%) | p-value | Covariate effect (RSE%) | p-value |
|  | Beta on $\delta$ | $-0.357 (32)$ | $0.002$ | $-0.38 (31)$ | $0.001$ |
|  | Gamma on $\theta$ | $8.39 (15)$ | $<{10}^{-6}$ | $7.9 (17)$ | $<{10}^{-6}$ |
|  | Delta on $p$ | $0.554 (50)$ | $0.047$ | $0.5(51)$ | $0.053$ |
|  | Delta on $\theta$ | $5.49 (15)$ | $<{10}^{-6}$ | $5.3 (28)$ | $3.4\times{10}^{-4}$ |
|  | Omicron on $p$ | $-2.82 (19)$ | $<{10}^{-6}$ | $-2.9 (18)$ | $<{10}^{-6}$ |
|  | Omicron on $\mu$ | $2.7 (23)$ | $1.98\times{10}^{-5}$ | $2.7 (20)$ | $<{10}^{-6}$ |
|  | Omicron on $\theta$ | $5.04 (18)$ | $<{10}^{-6}$ | $5.3 (28)$ | $4.3\times{10}^{-4}$ |
| *Criteria* | |  |  |  |  |
|  | BIC | 2360 | | 2360 | |
|  | -2LL | 2277 | | 2277 | |

4. More details are needed for the methodology:
a. how are the three data sets weighted?

Briefly, the same weight is given to each observation which is time, patient and marker specific. The general model is written as follows:

$$y_{ij}^{k}=f_{k}\left( t_{ij}, \psi_{i} \right)+\epsilon_{ij}^{k}$$

Where $y_{ij}^{k}$ is the $j^{th}$ observation of individual $i$ of biomarker $k$, and $\psi_{i}$ is the parameter vector of structural function $f$ for individual $i$. We assume a constant error term $\epsilon_{ij}^{k}$ of mean 0 and standard deviation $\sigma_{k}[16,17]$. As we have 3 biomarkers (genomic RNA, subgenomic RNA and infectious titers) they all can be written in the same way. We then have the following formula for the individual log-likelihood:

$$\mathcal{LL}\left( y_{i}^{1},y_{i}^{2},y_{i}^{3},\psi_{i} \right)=\sum_{j} \left[ p\left( y_{ij}^{1} | \psi_{i} \right)+p\left( y_{ij}^{2} | \psi_{i} \right)+p\left( y_{ij}^{3} | \psi_{i} \right) \right]+p\left( \psi_{i} \right|\theta)$$

In a Gaussian model we then have:

$$\mathcal{LL}\left( y^{1},y^{2},y^{3},\psi\right)=\sum_{j} -\log\left( \sqrt{2\pi}\sigma_{1} \right)-\sum_{j} \frac{\left( y_{ij}^{1}-f_{1}\left( t_{ij}, \psi_{i} \right) \right)^{2}}{2\sigma_{1}^{2}}+$$

$$=\sum_{j} -\log\left( \sqrt{2\pi}\sigma_{2} \right)-\sum_{j} \frac{\left( y_{ij}^{2}-f_{2}\left( t_{ij}, \psi_{i} \right) \right)^{2}}{2\sigma_{2}^{2}}+\sum_{j} -\log\left( \sqrt{2\pi}\sigma_{3} \right)-\sum_{j} \frac{\left( y_{ij}^{3}-f_{3}\left( t_{ij}, \psi_{i} \right) \right)^{2}}{2\sigma_{3}^{2}}$$

b. Which procedure was used for sensitivity?

All sensitivity analysis was performed by profile likelihood. We fixed the parameters in question to potential values to evaluate the impact of the parameter on both the parameter’s relative standard errors and the adequation to data using BIC criteria.

c. What determined the choice of distributions for different parameters, and are those choices biasing the results?

As we want to make our model as generalizable as possible we use basic distribution only to ensure certain conditions. Almost all our parameters follow a lognormal distribution to ensure positivity and only two parameters follow a logit-normal distribution (parameters $\mu$ and $h$) as they are proportion. We limit the assumptions made on the distribution of our parameter to avoid any form of bias.

5. All longitudinal data should be presented in the manuscript. Also, it is very hard to distinguish between squares and circles, maybe color (or filled objects) will make that easier to understand.

All animal data, statistical analysis code and modelling files were published at submission but the data availability statement was missing in the main manuscript.

All data and code used are available on Zenodo at link: <https://doi.org/10.5281/zenodo.7304183>

6. Why are some F effects density dependent? Have the authors investigate linear and power law terms?

We thank the reviewer for this comment as a mistake was made in the main manuscript. The formula for immune response model 3 (reduction of the infectivity rate $\beta$) should have been written as:

$$\frac{dT}{dt}=-\beta\left( 1-\frac{F_{20}}{F_{20}+\theta} \right)V_{I}T (34)$$

$$\frac{dI_{1}}{dt}=\beta\left( 1-\frac{F_{20}}{F_{20}+\theta} \right)V_{I}T-kI_{1} (35)$$

The underlying idea is that parameters are reduced by a certain percentage reflective of the immune response strength. This is done using Emax type of equations that bounds the immune response effect between 0 and 1. The immune response strength is then dictated by the $\theta$ parameter. On the contrary, in model 2 (increased infected cells clearance) the effect of the immune system can increase the parameter by several fold change, this is reflected by the $\varphi$ parameter in equation (33).

The results and methods have been updated accordingly.

**S1 Fig. Relationship between genomic RNA and infectious titers.** We represent the longitudinal values of genomic RNA for each individual and if the associated PFU sampe is detectable or not.

S5 Fig. **Impact of different inoculum on viral dynamic simulations.** Using simulations, we sampled parameters considering both the uncertainty in the estimation and the inter-individual variability (see methods). Only the mean viral load was shown for clarity.

**S6 Fig. Dynamics of the immune response.** We represent the simulation of our immune response model. For clarity we only show the last effector compartment (F20) for each variant. We used the population parameters to simulate the mean trajectory of each variant.

**S7 Fig. Dynamics of cytokines and correlation with viral load metrics.** A) Median concentration of measured cytokines. B) Correlation between AUC of viral load predicted by our model and the cytokine AUC. C) Correlation between peak viral load predicted by our model and peak cytokine concentration.

# References

1.	McMahan K, Yu J, Mercado NB, Loos C, Tostanoski LH, Chandrashekar A, et al. Correlates of protection against SARS-CoV-2 in rhesus macaques. Nature. 2021;590: 630–634. doi:10.1038/s41586-020-03041-6

2.	Moss P. The T cell immune response against SARS-CoV-2. Nat Immunol. 2022;23: 186–193. doi:10.1038/s41590-021-01122-w

3.	Chandrashekar A, Liu J, Martinot AJ, McMahan K, Mercado NB, Peter L, et al. SARS-CoV-2 infection protects against rechallenge in rhesus macaques. Science. 2020;369: 812–817. doi:10.1126/science.abc4776

4.	Chakraborty C, Sharma AR, Bhattacharya M, Lee S-S. A Detailed Overview of Immune Escape, Antibody Escape, Partial Vaccine Escape of SARS-CoV-2 and Their Emerging Variants With Escape Mutations. Frontiers in Immunology. 2022;13. Available: https://www.frontiersin.org/articles/10.3389/fimmu.2022.801522

5.	Haller O, Kochs G, Weber F. The interferon response circuit: Induction and suppression by pathogenic viruses. Virology. 2006;344: 119–130. doi:10.1016/j.virol.2005.09.024

6.	Thiel V, Weber F. Interferon and cytokine responses to SARS-coronavirus infection. Cytokine & Growth Factor Reviews. 2008;19: 121–132. doi:10.1016/j.cytogfr.2008.01.001

7.	Shalamova L, Felgenhauer U, Wilhelm J, Schaubmar AR, Büttner K, Schoen A, et al. Omicron variant of SARS-CoV-2 exhibits an increased resilience to the antiviral type I interferon response. PNAS Nexus. 2022;1: pgac067. doi:10.1093/pnasnexus/pgac067

8.	Guo K, Barrett BS, Morrison JH, Mickens KL, Vladar EK, Hasenkrug KJ, et al. Interferon resistance of emerging SARS-CoV-2 variants. Proceedings of the National Academy of Sciences. 2022;119: e2203760119. doi:10.1073/pnas.2203760119

9.	Lista MJ, Winstone H, Wilson HD, Dyer A, Pickering S, Galao RP, et al. The P681H Mutation in the Spike Glycoprotein of the Alpha Variant of SARS-CoV-2 Escapes IFITM Restriction and Is Necessary for Type I Interferon Resistance. Journal of Virology. 2022;96: e01250-22. doi:10.1128/jvi.01250-22

10.	Gonçalves A, Maisonnasse P, Donati F, Albert M, Behillil S, Contreras V, et al. SARS-CoV-2 viral dynamics in non-human primates. PLOS Computational Biology. 2021;17: e1008785. doi:10.1371/journal.pcbi.1008785

11.	Sender R, Bar-On YM, Gleizer S, Bernshtein B, Flamholz A, Phillips R, et al. The total number and mass of SARS-CoV-2 virions. PNAS. 2021;118. doi:10.1073/pnas.2024815118

12.	Maisonnasse P, Aldon Y, Marc A, Marlin R, Dereuddre-Bosquet N, Kuzmina NA, et al. COVA1-18 neutralizing antibody protects against SARS-CoV-2 in three preclinical models. Nat Commun. 2021;12: 6097. doi:10.1038/s41467-021-26354-0

13.	Alexandre M, Marlin R, Prague M, Coleon S, Kahlaoui N, Cardinaud S, et al. Modelling the response to vaccine in non-human primates to define SARS-CoV-2 mechanistic correlates of protection. Graw F, Davenport MP, editors. eLife. 2022;11: e75427. doi:10.7554/eLife.75427

14.	Néant N, Lingas G, Hingrat QL, Ghosn J, Engelmann I, Lepiller Q, et al. Modeling SARS-CoV-2 viral kinetics and association with mortality in hospitalized patients from the French COVID cohort. PNAS. 2021;118. doi:10.1073/pnas.2017962118

15.	Gonçalves A, Bertrand J, Ke R, Comets E, de Lamballerie X, Malvy D, et al. Timing of antiviral treatment initiation is critical to reduce SARS‐CoV‐2 viral load. CPT Pharmacometrics Syst Pharmacol. 2020; psp4.12543. doi:10.1002/psp4.12543

16.	Comets E, Lavenu A, Lavielle M. Parameter Estimation in Nonlinear Mixed Effect Models Using saemix, an R Implementation of the SAEM Algorithm. Journal of Statistical Software. 2017;80: 1–41. doi:10.18637/jss.v080.i03

17.	Kuhn E, Lavielle M. Maximum likelihood estimation in nonlinear mixed effects models. Computational Statistics & Data Analysis. 2005;49: 1020–1038. doi:10.1016/j.csda.2004.07.002
