# Supplementary material for: Impact of variants of concern on SARS-CoV-2 viral dynamics in non-human primates
Source: PLoS Comput Biol. 2023 Aug 9;19(8):e1010721. doi: 10.1371/journal.pcbi.1010721 (PMC10441782; doi:10.1371/journal.pcbi.1010721)
Supplement: Attachment — Submitted filename: AnswerToReviewers - Plos.docx [file pcbi.1010721.s012.docx]

# Impact of variants of concern on SARS-CoV-2 viral dynamics in non-human primates

Aurélien Marc^1^, Romain Marlin^2^, Flora Donati^3,4^, Mélanie Prague^5,6^, Marion Kerioui^1^, Cécile Hérate^2^, Marie Alexandre^5,6^ , Nathalie Dereuddre-bosquet^2^, Julie Bertrand^1^, Vanessa Contreras^2^, Sylvie Behillil^3,4^, Pauline Maisonnasse^2^, Sylvie Van Der Werf ^3,4^, Roger Le Grand^2^, Jérémie Guedj^1^

1 Université Paris Cité, INSERM, IAME, UMR 1137, 75018, Paris, France

2 Université Paris-Saclay, Inserm, CEA, Center for Immunology of Viral, Auto-immune, Hematological and Bacterial diseases (IMVA-HB/IDMIT), Fontenay-aux-Roses and Le Kremlin-Bicêtre, Paris, France

3 National Reference Center for Respiratory Viruses, Institut Pasteur, 28 rue du Docteur Roux, 75015 Paris, France.

4 Molecular Genetics of RNA Viruses Unit, Institut Pasteur, UMR3569, CNRS, Université de Paris, 28 rue du Docteur Roux, 75015 Paris, France.

5 Inria Bordeaux Sud-Ouest, Inserm, Bordeaux Population Health Research Center, SISTM Team, UMR 1219, University of Bordeaux, Bordeaux, France

6 Vaccine Research Institute, Créteil, France

**Responses to referees and editor**

Reviewer #1:

Regarding my previous comment (Comment 1), the authors hypothesized in their revision that the immune response modeled in this mathematical model would predominantly favor the effect of the innate immune response, which serves as the host's first line of defense during an infection. However, the authors were unable to identify a clear correlation between the model-predicted immune response (F20) in S6 Fig and the measured cytokines (IL15, IL1ra, MCP1) in S7 Fig, as they did not measure IFN responses. Notably, the measured cytokine peaks occur around 2 dpi, while the model prediction peak occurs after 5 dpi. This discrepancy arises because the authors assumed a delay of 3 days in the antigen-mediated immune induction (Eqs. 11-30), which implies that the peak cannot occur before 3 dpi. If the authors wish to reference the measured cytokine dynamics, they must relax the assumption of fixed parameters in the antigen-mediated immune induction process of Model (1), specifically j, tau, and d_F. Otherwise, I fail to comprehend the purpose of including and comparing S6 Fig and S7A Fig as conducted by the authors, and I anticipate that validating their hypothesis would be challenging even with the availability of IFN data (which may peak earlier than 5 dpi). Thus, it is necessary for the authors to perform a sensitivity analysis regarding the fixed parameters.

We acknowledged the reviewer’s remark and performed a sensitivity analysis on the different parameters of the immune response model (i.e. parameters $j$ and $\tau$, the number of transitioning compartments and mean time spent in those compartments respectively). We tested values for $j$ ranging from 5 to 30 compartments and for $\tau$ ranging from 1 to 6 days spent in those compartments. This allows us to modulate the mean delay in the immune response and its distribution. Across all simulation, we found that our model consistently predicts a peak concentration occurring more than 4 days post infection (S3 Table). This shows that the peak of the immune response predicted by our model is not a consequence of the assumptions made on the kinetics of immune response, but rather a feature of the data. Nonetheless, having a peak of immune response at day 4 or more does not mean that the immune response is negligible until then. In fact, we extended S6 Fig with the dynamics of the infectious ratio parameter ($\mu$) over time (S6B Fig). As can be seen, even though the predicted kinetics of immune response peaks at day 6, the infectious ratio is already largely reduced by day 3.

We modified the methods, results and discussion to account for this limitation:

Discussion: “*This effect on the infectious ratio could be attributable to an effect of the innate response, in particular the interferon (IFN) response, that can confer antiviral, antiproliferative and immunomodulatory functions* [1]*. Our model also identified that the effects of the immune response was variant-dependent (captured by the covariates on the parameter* $\theta$ *in the model) consistent with the observations that SARS-CoV-2 variants exhibits mutations in nucleocapsid, membrane and non-structural proteins that antagonize IFN signalling in cells* [2,3]*. Unfortunately, this hypothesis remains speculative as IFN was not measured in this study, and the cytokines that were measured (e.g. MCP1, IL15 and IL1RA) did not correlate with the viral kinetics (S7 Fig). Of note, these cytokines peaked at day 2, i.e., earlier than predicted by our model which assumes a peak of the immune response by day 6 (S6 Fig). This could be due to the fact that these cytokines were measured in plasma, and not in the site of infection, but we nevertheless tested the sensitivity of this prediction to our hypotheses. For that purpose, we conducted a sensitivity analysis on the kinetics of the immune response by assuming different number of compartments (*$j$*) and mean time spent in those compartments (*$\tau)$*, allowing to modulate the mean delay of immune response and its distribution. Interestingly, in all simulations, the model predicted a peak of the immune response later than day 4. Using the final model, we nevertheless show that the impact of the immune response in reducing the infectious ratio is already significant at day 3 post infection S6B Fig).”*

Results: “*To challenge our model assumption on the immune response compartment, we performed a sensitivity analysis on the number of transitioning compartments (*$j$*) and the mean time spent in those compartments (*$\tau$*). We also performed the covariate selection on all models. We found that largely similar VoC-specific covariates were selected regardless of the delay in the immune response (S3 and S4 Fig). In addition, we found that the model assuming a time spent of 3 days in the transitioning compartment yielded the best results (S3 Table).”*

Methods: “*To allow some variability in this response we assumed a certain production phase before the immune response takes effect. We introduced this delayed effector compartment using the Linear Chain Trick (LCT)* [4] *by modelling a successive number of transitioning compartments. This assumes an Erlang distribution with parameters* $j$ *and* $\tau$ *representing the number of transitioning compartments and the mean time spent in those compartments respectively. We fixed those parameters to* $j=20$ *compartments and* $\tau=3$ *days to account for the setup of the immune response. We then performed a sensitivity analysis on those parameters varying both the number of compartments (from 5 to 30) and the mean time spent in those compartments (from 1 to 6 days).”*

**S6 Fig. Dynamics of the immune response and its effect.** A) Median trajectory of the last compartment of our immune response B) Median trajectory of the infectious ratio parameter $\mu$ over time. We used the population parameters of our best model to simulate the median trajectory of each variant.

**S3 Table. Sensitivity analysis on the delayed immune response.** Using the best structural model (i.e. Model 1 including an effect on the infectious ratio) we tested several delays for the immune response to take place and performed the covariate search algorithm on all models.

| Model ID | $j$  Number of compartments | $\tau$  Mean time spent | $g=\frac{j}{\tau}$  Transfer rate | Time to peak of immune compartment  (days) | BIC before COSSAC | BIC after COSSAC (ΔBIC ) |
| --- | --- | --- | --- | --- | --- | --- |
| 1 | 5 | 1 | 5 | 4 | 2451 | 2429 (-22) |
| 2 | 5 | 2 | 2.5 | 5 | 2409 | 2384 (-25) |
| 3 | 5 | 3 | 1.67 | 6 | 2405 | 2374 (-31) |
| 4 | 5 | 4 | 1.25 | 6 | 2408 | 2373 (-35) |
| 5 | 5 | 5 | 1 | 7 | 2408 | 2375 (-33) |
| 6 | 5 | 6 | 0.83 | 8 | 2409 | 2379 (-30) |
| 7 | 10 | 1 | 10 | 4 | 2432 | 2409 (-23) |
| 8 | 10 | 2 | 5 | 5 | 2409 | 2373 (-36) |
| 9 | 10 | 3 | 3.33 | 6 | 2410 | 2361 (-49) |
| 10 | 10 | 4 | 2.5 | 7 | 2411 | 2381 (-30) |
| 11 | 10 | 5 | 2 | 8 | 2413 | 2366 (-47) |
| 12 | 10 | 6 | 1.67 | 9 | 2414 | 2367 (-47) |
| 13 | 20 | 1 | 20 | 4 | 2426 | 2402 (-24) |
| 14 | 20 | 2 | 10 | 5 | 2409 | 2363 (-46) |
| 15 | 20 | 3 | 6.67 | 6 | 2411 | 2360 (-51) |
| 16 | 20 | 4 | 5 | 7 | 2414 | 2377 (-37) |
| 17 | 20 | 5 | 4 | 8 | 2416 | 2379 (-37) |
| 18 | 20 | 6 | 3.33 | 9 | 2417 | 2381 (-36) |
| 19 | 30 | 1 | 30 | 4 | 2424 | 2397 (-27) |
| 20 | 30 | 2 | 15 | 5 | 2408 | 2377 (-31) |
| 21 | 30 | 3 | 10 | 6 | 2413 | 2385 (-28) |
| 22 | 30 | 4 | 7.5 | 7 | 2417 | 2363 (-54) |
| 23 | 30 | 5 | 6 | 8 | 2419 | 2380 (-39) |
| 24 | 30 | 6 | 5 | 9 | 2420 | 2393 (-27) |

1.	Thiel V, Weber F. Interferon and cytokine responses to SARS-coronavirus infection. Cytokine & Growth Factor Reviews. 2008;19: 121–132. doi:10.1016/j.cytogfr.2008.01.001

2.	Shalamova L, Felgenhauer U, Wilhelm J, Schaubmar AR, Büttner K, Schoen A, et al. Omicron variant of SARS-CoV-2 exhibits an increased resilience to the antiviral type I interferon response. PNAS Nexus. 2022;1: pgac067. doi:10.1093/pnasnexus/pgac067

3.	Guo K, Barrett BS, Morrison JH, Mickens KL, Vladar EK, Hasenkrug KJ, et al. Interferon resistance of emerging SARS-CoV-2 variants. Proceedings of the National Academy of Sciences. 2022;119: e2203760119. doi:10.1073/pnas.2203760119

4.	Hurtado PJ, Richards C. Building mean field ODE models using the generalized linear chain trick & Markov chain theory. Journal of Biological Dynamics. 2021;15: S248–S272. doi:10.1080/17513758.2021.1912418
